# Functional characterization of acid-sensing ion channels in the cerebellum-originating medulloblastoma cell line DAOY and in cerebellar granule neurons

**DOI:** 10.1007/s00424-023-02839-3

**Published:** 2023-07-20

**Authors:** Karolos-Philippos Pissas, Maria Schilling, Yuemin Tian, Stefan Gründer

**Affiliations:** Institute of Physiology, RWTH Aachen University, Pauwelsstraße 30, 52074 Aachen, Germany

**Keywords:** Cell death, Extracellular acidity, Necroptosis, Tumour microenvironment, Tumour spheroids

## Abstract

Acid-sensing ion channels (ASICs) are Na^+^ channels that are almost ubiquitously expressed in neurons of the brain. Functional ASIC1a is also expressed in glioblastoma stem cells, where it might sense the acidic tumor microenvironment. Prolonged acidosis induces cell death in neurons and reduces tumor sphere formation in glioblastoma via activation of ASIC1a. It is currently unknown whether ASICs are expressed and involved in acid-induced cell death in other types of brain tumors. In this study, we investigated ASICs in medulloblastoma, using two established cell lines, DAOY and UW228, as in vitro models. In addition, we characterized ASICs in the most numerous neuron of the brain, the cerebellar granule cell, which shares the progenitor cell with some forms of medulloblastoma. We report compelling evidence using RT-qPCR, western blot and whole-cell patch clamp that DAOY and cerebellar granule cells, but not UW228 cells, functionally express homomeric ASIC1a. Additionally, Ca^2+^-imaging revealed that extracellular acidification elevated intracellular Ca^2+^-levels in DAOY cells independently of ASICs. Finally, we show that overexpression of RIPK3, a key component of the necroptosis pathway, renders DAOY cells susceptible to acid-induced cell death via activation of ASIC1a. Our data support the idea that ASIC1a is an important acid sensor in brain tumors and that its activation has potential to induce cell death in tumor cells.

## Introduction

Brain tumors constitute the second most common type of cancer in children just behind leukemia, and medulloblastoma (MB) is the most common pediatric brain tumor [53]. MB is heterogeneous; the WHO classifies MB into 5 different histological groups and 4 molecular subgroups [43]. Individual prognosis varies strongly, depending on the underlying histopathological and molecular subgroup of the tumor [54]. Children suffering from MB driven by Wingless signaling (WNT MB, molecular subgroup 1, 10% of all MB) have the best prognosis with a survival rate of > 95% during a 5 year interval. Patients with MB driven by sonic hedgehog signaling (SHH MB, 30% of all MB) also have a relatively good prognosis. On the other hand, patients with group 3 MB (30% of all MB) have the worst outcome with a survival rate of < 60% during a 5 year interval [34]. Due to drastic treatment improvements over the recent decades, MB has become, in many cases and depending on the subgroup, a manageable disease. However, these improvements have been primarily achieved by employing very aggressive treatment regimens including chemotherapy, craniospinal radiation and surgery [34]. In turn, such aggressive treatments cause severe adverse effects in the developing child, for instance by permanently impairing neurocognitive functions, reducing growth and inducing endocrine dysfunctions [31, 38, 48]. Therefore, further improvements cannot be achieved by increasing the intensity of already existing treatment options. Additional treatment and better prognosis for children suffering from MB can only be achieved by uncovering novel mechanisms and targets, tailored, ideally, to the individual tumor’s cellular and molecular profile.

In recent years, it has increasingly been recognized that an acidic pH is a hallmark of the tumor microenvironment (TME). The acidic TME is caused by the Warburg effect, high proliferation rates and inadequate tumor vascularization. It typically modulates invasiveness, proliferation, cell death and stemness of tumors [1, 3, 35, 41]. Therefore, the identification of acid sensors on the surface of tumor cells is necessary to increase our understanding of the many effects of an acidic TME on tumors. Previously, acid-sensitive K2P K^+^ channels TASK-1 and TASK-3 [15] and the acid-sensitive G protein-coupled receptors (GPCRs) OGR1 and G2A [28] have been investigated in DAOY cells, a relatively common cellular model for SHH MB [29].

Acid-sensing ion channels (ASICs) are moderately selective Na^+^ channels that are directly opened by a drop in extracellular pH and are important neuronal acid sensors. In the central nervous system (CNS), three ASIC subunits assemble into functional hetero- or homotrimeric channels: ASIC1a, ASIC2a and ASIC2b [4, 7, 42, 46]. Concerning tumor tissue, it has been reported that ASIC1a is highly upregulated in breast cancer patients and that its downregulation decreased proliferation and migration in vitro and in vivo [63]. ASIC2 is expressed in colorectal cancer where it increases invasiveness, while ASIC1 and ASIC3 may play a pivotal role in the epithelial-mesenchymal transition (EMT) of pancreatic cancer [66, 67]. Functional homomeric ASIC1a is also expressed in glioblastoma stem cell lines (GSCs) [55]. While ASICs do not contribute to the aggressive migration of GSCs in vitro [9], activation of ASIC1a reduces tumor sphere formation of GSCs by inducing a Nec1-dependent cell death that is related to but not identical with necroptosis [8]. Nec-1 is an inhibitor of receptor interacting protein kinase 1 (RIPK1) and it had previously been demonstrated that ASIC1a activation induces a RIPK1-dependent cell death mechanism in neurons, which could contribute to the neurodegeneration accompanying ischemic stroke [58]. The canonical necroptosis pathway involves a signaling cascade of RIPK1, RIPK3 and mixed lineage kinase domain-like protein (MLKL) [24]. However, while ASIC1a-mediated cell death in GSCs depends on RIPK1 and RIPK3, it is independent of MLKL [8]. Whether ASICs are functional and whether they can induce cell death in MB cells is unknown.

MB cells of the SHH subtype originate from granule cell progenitors (GCPs) [19]. Therefore, it is of interest to compare ASICs of SHH MB cells, such as DAOY cells, with cerebellar granule cells (CGCs), with which they share the same progenitor cell [25, 50]. Although CGCs are the most numerous neurons in the brain, data on ASIC expression and function in CGCs is currently sparse [16, 33].

Here, we characterized ASICs in two MB cell lines, DAOY and UW228 [30, 36], and in primary CGCs from 7-day-old mice. We show that DAOY and CGCs, but not UW228, express ASIC1a and ASIC2 mRNA and functionally express mainly homomeric ASIC1a. In addition, we show that extracellular acidification elicits a RIPK1/3-dependent cell death in DAOY cells.

## Results

### DAOY cells express functional ASIC1a, while UW228 cells do not express functional ASICs

**Fig. 1:**
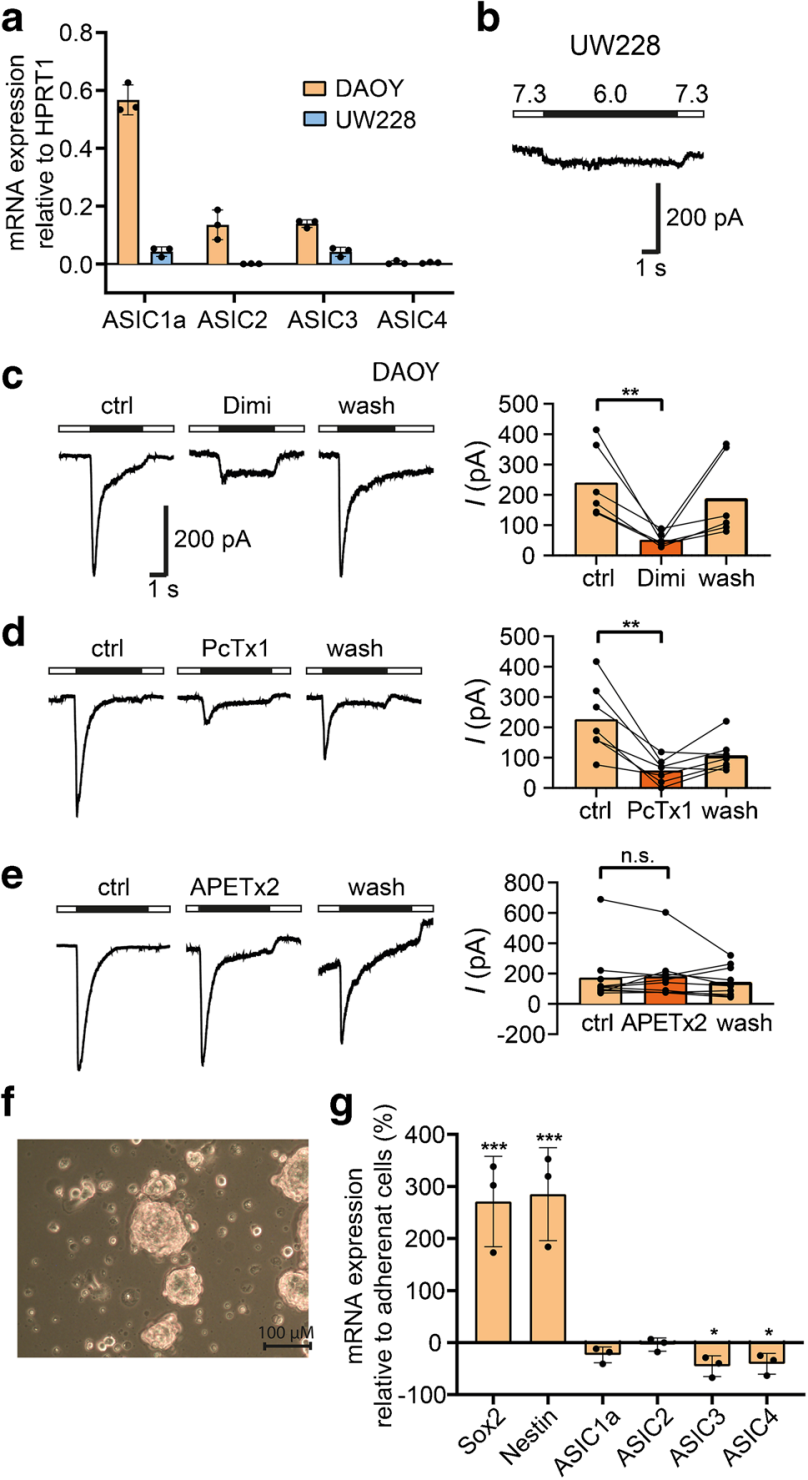
DAOY cells express functional ASIC1a, while UW228 cells do not. **a** mRNA expression of *ASIC1a*, *ASIC2a/b*, *ASIC3*, and *ASIC4* in adherent DAOY and UW228 monolayers normalized to *HPRT1* expression levels (*n* = 3). **b** Example trace of the current elicited with a pH 6.0 stimulation in an adherent UW228 cell. **c** Left, example traces of an ASIC current in an adherent DAOY cell elicited with a pH 6.0 stimulation (ctrl) and inhibited with 30 μM diminazene (Dimi). Right, summary of current amplitudes at -70 mV (*n* = 6). The conditioning pH between different stimuli was 7.3. **d** as in c but with 100 nM PcTx1 (*n* = 7). **(e)** as in c but with 500 nM APETx2 (*n* = 10; n.s., no statistical significance). **f** Light microscopy images of DAOY medullospheres cultured for 7 days. **g** mRNA expression of *Sox2*, *Nestin*, *ASIC1*-*ASIC4* in DAOY medullospheres compared to adherent DAOY cells (*n* = 3). Results are shown as mean ± SD. Statistical analysis was performed with a one-way ANOVA (**g**) or paired Student’s t-tests (**c–e**). *, *P* < 0.05; **, *P* < 0.01; ***, *P* < 0.001

First, we determined the relative expression of the *ASIC1a*, *ASIC2a/b*, *ASIC3* and *ASIC4* genes in DAOY and UW228 cells using RT-qPCR. DAOY cells are a model for SHH MB and UW228 cells for WNT MB [26]. We used the housekeeping gene *HPRT1* as a reference gene [10]. DAOY cells expressed *ASIC1a* most abundantly, followed by a fourfold lower expression of *ASIC2a/b* and *ASIC3* (Fig. 1a). In contrast, UW228 cells expressed all ASIC genes at very low levels (Fig. 1a).

Next, we characterized ASIC-currents in MB cells using the whole-cell patch clamp method. Cells were kept in a bath solution with a conditioning pH of 7.3. Application of pH 6.0 elicited a typical transient ASIC current in almost 90% of DAOY cells (Fig. 1c), but not in UW228 cells (Fig. 1b), which is in accordance with the measured mRNA levels of ASIC subunits in the two cell lines (Fig. 1a). The current in DAOY cells was inhibited by the unspecific ASIC pore blocker diminazene [49] (Fig. 1c), confirming that it was mediated by ASICs. We further determined the identity of these ASICs by applying PcTx1, a specific inhibitor of homomeric ASIC1a, and APETx2, a specific inhibitor of ASIC3-containing channels. While the ASIC current was almost fully inhibited by PcTx1 (Fig. 1d), APETx2 had no effect (Fig. 1e), suggesting that homomeric ASIC1a was the predominant ASIC in DAOY cells.

We also determined whether the expression of ASICs would be altered in a stem cell-enriched environment. To this end, DAOY cells were cultured as medullospheres (MBS) in suspension, as previously described [21] (Fig. 1f). The increased stemness of MBS cultures was confirmed by a threefold increased expression of the stem cell markers *Sox2* and *Nestin* compared to adherent DAOY cells (Fig. 1g). While *ASIC1a* expression only slightly decreased (*p* = 0.241), *ASIC3* (*p* = 0.022) and *ASIC4* (*p* = 0.036) expression decreased two-fold in the MBS culture. Since ASIC expression did not greatly change in a stem cell enriched culture, we continued our study with adherent DAOY cells.

### Primary mouse CGCs express functional ASIC1a

**Fig. 2:**
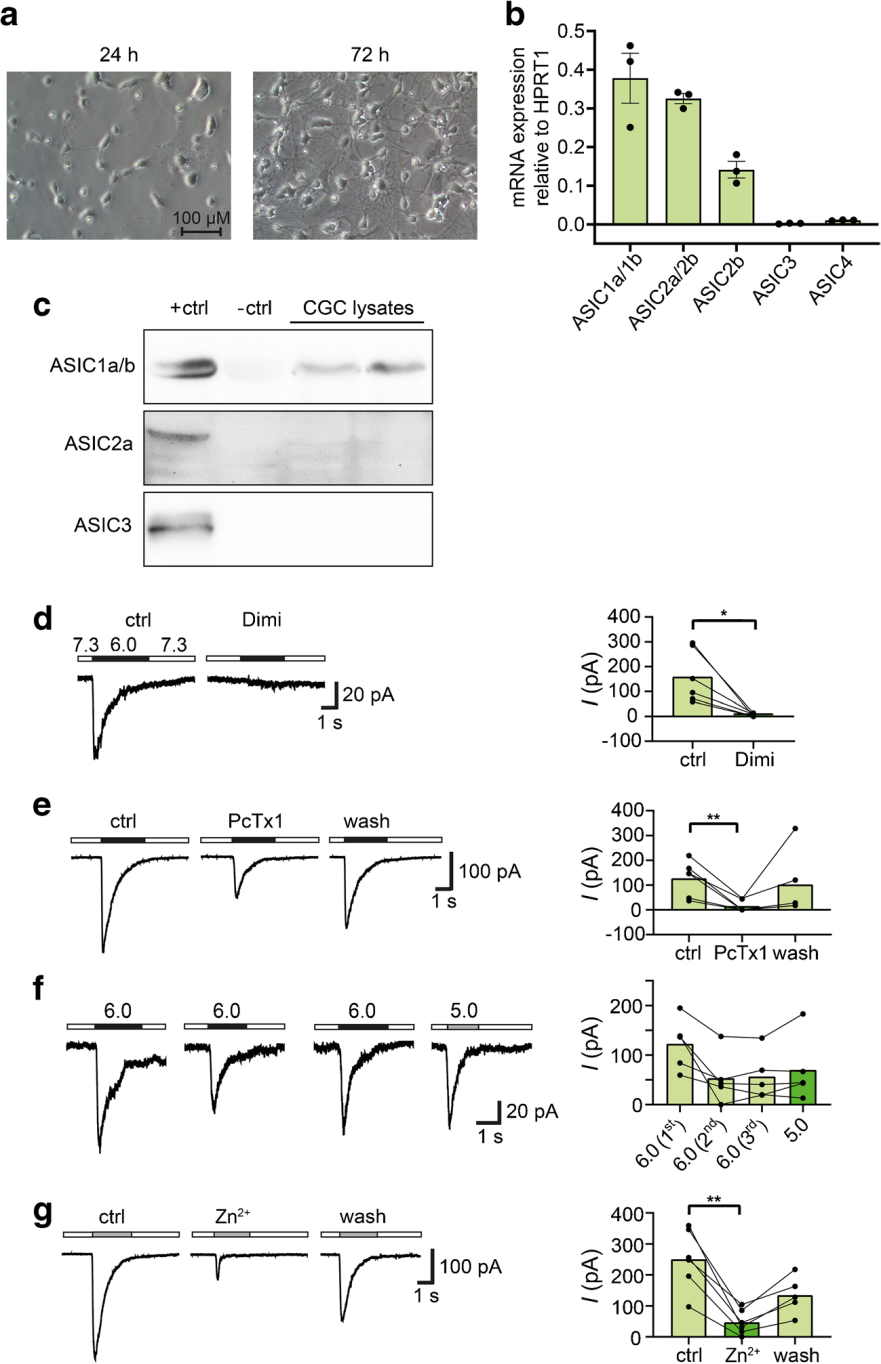
Mouse CGCs cultured for 24 – 72 h express functional ASIC1a. **a** Light microscopy images of CGCs cultured for 24 h and 72 h, respectively. **b** mRNA expression of *ASIC1a/b*, *ASIC2a/b*, *ASIC2b*, *ASIC3* and *ASIC4* in CGCs cultured for 72 h was determined by qPCR (*n* = 3). **c** Immunoblots for ASIC1, ASIC2a and ASIC3 in mouse CGCs (*n* = 2). **d** Left, representative traces of the effect of 30 μM diminazene (Dimi) on the ASIC-currents elicited by pH 6.0. Conditioning pH was 7.3. Right, bar graphs summarizing current amplitudes (*n* = 6). **e** as in **d** but with 100 nM PcTx1. **f** Left, representative traces of ASIC currents elicited by pH 6 or pH 5 in the same cell. Right, bar graphs summarizing current amplitudes (*n* = 5). **g** as in **d** but with 200 μM Zn^2+^. ASIC currents were elicited with pH 5.0 (*n* = 6). Results are shown as mean ± SD (**b**, **d**-**g**). Statistical analysis was performed with paired Student’s t-tests (**d**-**g**). *, *P* < 0.05; **, *P* < 0.01

We used mouse CGCs as a model to study the expression of ASICs in CGCs. CGCs were isolated and cultured from 7 day old wild type (WT) mice. They started extending neurites only a few hours after being put into culture (Fig. 2a). RT-qPCR analysis revealed expression of *ASIC1a/b* and *ASIC2a/b* genes (Fig. 2b). When normalized to *HPRT*, the expression of *ASIC1a/b* was comparable to the expression in DAOY cells; the expression of *ASIC2a/b* in mouse CGCs was approximately two-fold higher, which is consistent with a generally higher abundance of ASIC2a in mouse compared to human brain [11, 39]. RT-qPCR using isoform-specific primers revealed that about two-thirds of the *ASIC2* transcripts were *ASIC2a* and about one-third were *ASIC2b*. *ASIC3* and *ASIC4* mRNA were not expressed. While we could detect ASIC1a protein in CGCs using western blot (Fig. 2c), ASIC2a and ASIC3 proteins were not detected (*n* = 2), suggesting lower abundance of ASIC2a than ASIC1a. For western blots, we used HEK-293 overexpressing mouse ASIC1a, ASIC2a or ASIC3 as positive controls, and CHO cells, which do not express ASICs, as negative controls.

**Fig. 3:**
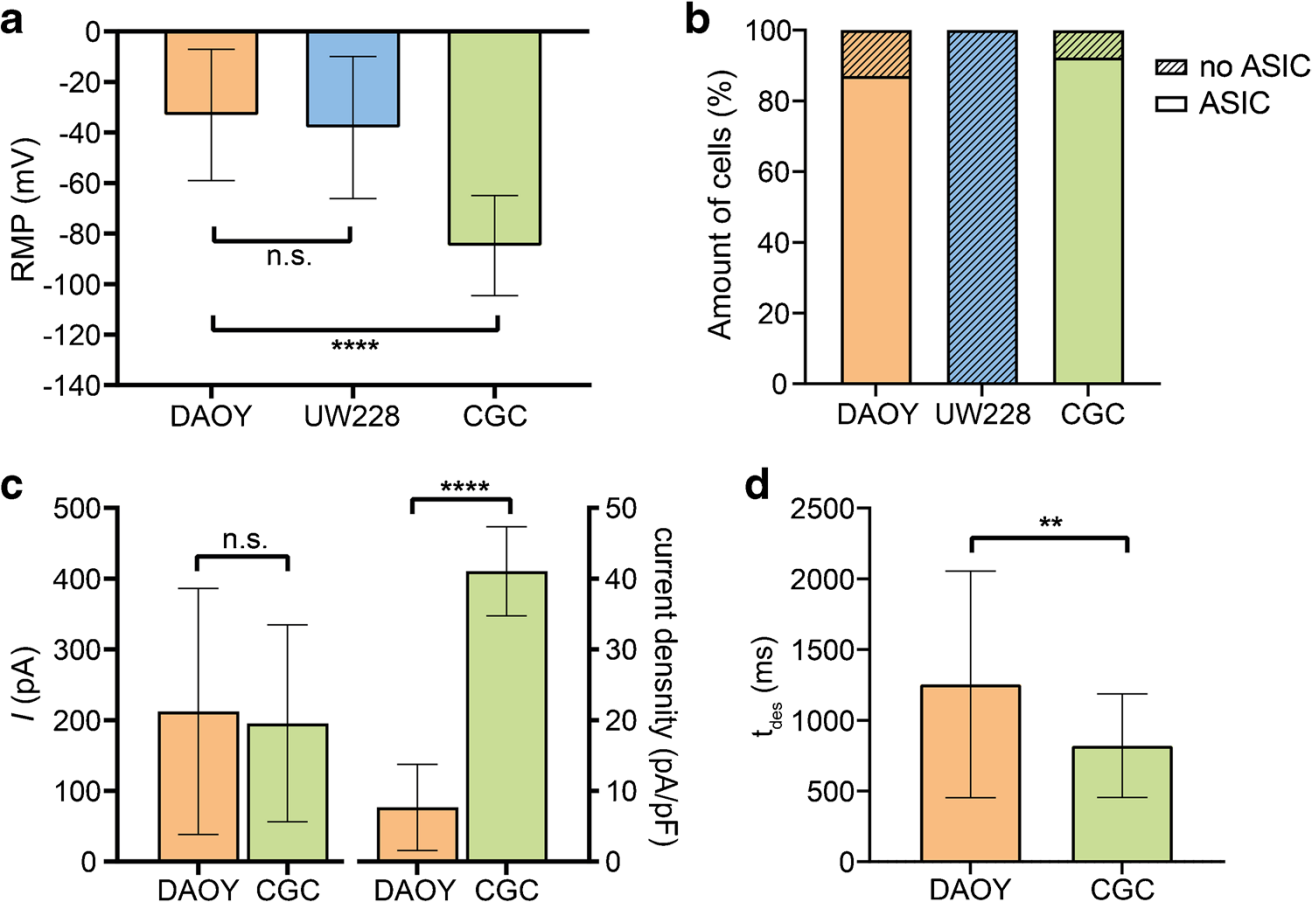
Comparison of RMP and ASIC currents between DAOY cells, UW228 cells and CGCs. **a** Summary of RMP in DAOY (*n* = 413), UW228 (*n* = 75), and CGCs (*n* = 64). **b** Relative amount of DAOY cells and CGCs that had a typical ASIC current. **c** Summary of current amplitude and current density in DAOY cells (*n* = 26) and CGC (*n* = 33). **d** Summary of desensitization time constant τ in DAOY cells (*n* = 26) and CGCs (*n* = 33**)**. The data used in this figure were taken from all patch clamp experiments, including those shown in Figs. 1 and 2. Results are shown as mean ± SD (**a**, **c**, **d**). Statistical analysis was performed with unpaired Student’s t-tests (**a**, **c**, **d**). n.s., no statistical significance; **, *P* < 0.01; ****, *P* < 0.0001

Whole-cell patch clamp experiments confirmed the functional expression of ASICs in mouse CGCs. pH 6.0 elicited ASIC-like currents in > 90% of the cells (see below; Fig. 3b). These currents were efficiently inhibited by diminazene (Fig. 2d) and by PcTx1 (Fig. 2e), confirming that the current was mainly mediated by ASIC1a homomers. To further investigate the presence of ASIC1a/2a heteromers, we used a stronger acidic stimulation (pH 5.0), since these channels are less sensitive to H^+^ than ASIC1a homomers or ASIC1a/2b heteromers. Stimulation with pH 5.0, however, did not elicit larger currents than pH 6.0 (Fig. 2f). Moreover, we pre- and coapplied 200 μM Zn^2+^ with pH 5.0. This strongly decreased the ASIC current amplitude (Fig. 2g). Because Zn^2+^ decreases current amplitude of ASIC1a homomers but increases the amplitude of ASIC1a/2a heteromers, [2, 6] these results further confirm that homomeric ASIC1a is the main ASIC of mouse CGCs.

### ASIC currents in DAOY cells have similar amplitudes than in CGCs, but smaller densities and slower desensitization

Next, we compared the resting membrane potential (RMP) of DAOY cells, UW228 cells and CGCs in the current clamp mode of the whole-cell patch clamp method (Fig. 3a). While the RMP was strongly depolarized to -32.32 ± 26.33 mV in DAOY und UW228 cells, which is a pathological characteristic of malignant cells and promotes proliferation and migration [64], CGCs had a much more negative RMP of -84.79 ± 19.79 mV, typical of mature neurons capable of generating action potentials (APs). As already observed, the majority (> 85%) of DAOY cells and CGCs produced an ASIC current during a pH 6.0 stimulation, while none of the UW228 cells did (Fig. 3b). The amplitude of ASIC currents varied from 31.2 pA to 689.5 pA with a mean of 212.3 ± 173.9 pA in DAOY cells and from 47.1 pA to 618.6 pA with a mean of 195.4 ± 139.2 pA in CGCs, respectively. The current density of CGCs was about 4- to 5-fold larger (Fig. 3c), due to the small capacitance of CGCs, as they are among the smallest neurons of the CNS. Furthermore, ASIC currents desensitized more quickly (*p* = 0.0076) in CGCs (τ = 820.3 ± 366.8 ms) than in DAOY cells (τ = 1254 ± 801.0 ms) (Fig. 3d).

### Extracellular acidification increases intracellular Ca^2+^ -levels in DAOY cells, but not in CGCs

**Fig. 4:**
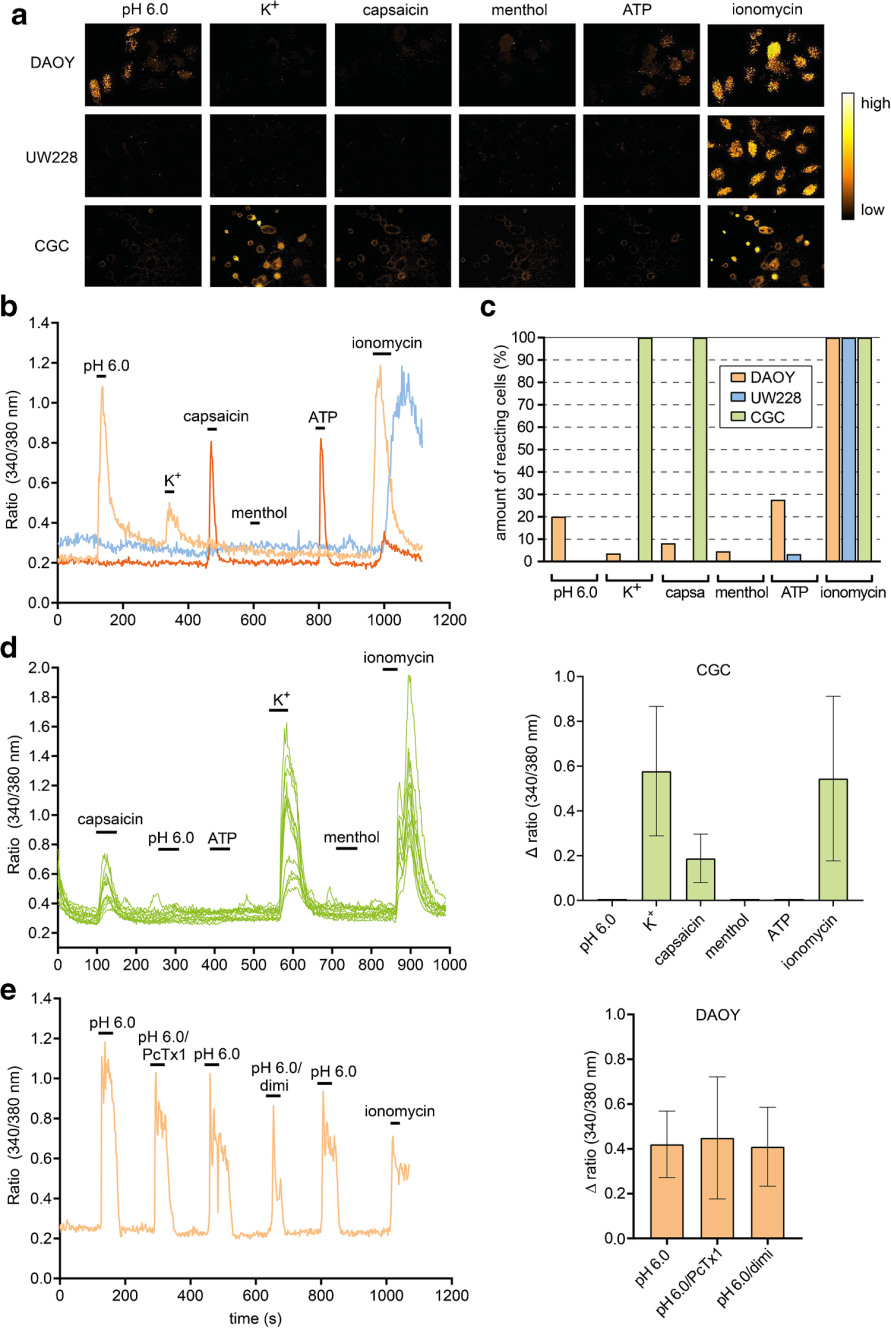
Ca^2+^-responses of DAOY cells, UW228 cells, and CGCs to different stimuli. **a** Representative pseudo-colored images of Ca^2+^-responses in DAOY cells, UW228 cells, and mouse CGCs to different stimuli. **b** Example Ca^2+^-responses of two DAOY cells (orange) and one UW228 cell (blue) to different stimuli. **c** Relative amount of DAOY cells, UW228 cells and CGCs responding to different stimuli with an increase in intracellular Ca^2+^-level. **d** Left, example Ca^2+^-responses of CGCs to different stimuli. Right, summary data (*n* = 58). **e** Left, example Ca^2+^-response of a DAOY cell to pH 6.0 in the absence or presence of 100 nM PcTx1 or 30 μM diminazene (dimi). Ionomycin was used as a positive control. Right, summary data. pH 6.0: *n* = 19; 6.0/PcTx1: *n* = 17; 6.0/dimi: *n* = 34. Results in bar graphs represent mean ± SD (**c**, **d**). Statistical analysis in panel d was performed with a one-way ANOVA

ASIC1a homomers are Na^+^ channels but also have a low Ca^2+^ permeability [32, 56, 57]. To investigate whether extracellular acidification elevates intracellular Ca^2+^-levels via homomeric ASIC1a in DAOY cells, UW228 cells and CGCs, we performed ratiometric Ca^2+^-imaging. The cells were loaded with Fura-2 and stimulated with different extracellular solutions containing either pH 6.0, high K^+^ (30 mM), 100 μM capsaicin, 200 μM menthol, 100 μM ATP, or 1 μM of the Ca^2+^ ionophore ionomycin as a positive control (Fig. 4a, b). DAOY cells responded quite broadly but not uniformly to the different stimuli (Fig. 4a-c). pH 6.0 elicited a Ca^2+^ signal in 20% of the cells (total of 337 cells) and ATP in 28%. < 10% of the cells reacted to capsaicin, menthol or high K^+^ solutions, suggesting the expression of TRPV1-, TRMP8- and voltage-gated Ca^2+^-channels (VGCCs), respectively, in small subpopulations of DAOY cells (Fig. 4c). In contrast to DAOY cells, only a few UW228 cells reacted to ATP (7 out of 316) and none reacted to the other stimuli. Because high K^+^ concentrations elicited Ca^2+^-signals in only a small subpopulation (5–10% of cells) of the immature and progenitor-cell like DAOY and in none of the UW228 cells, either most of these cells expressed no or few VGCCs or, at their depolarized RMP (Fig. 3a), most of the VGCCs in these cells were inactivated and unable to respond to high K^+^ concentrations.

CGCs responded much more uniformly than MB cells to the stimuli (Fig. 4d). pH 6.0 did not elicit a Ca^2+^-signal in any CGC, indicating that the Ca^2+^-permeability of ASIC1a homomers is too low and that the acid-sensitive GPCR OGR1 is inhibited by the Ca^2+^-sensing receptor CaSR, as previously described [28]. In contrast, high K^+^ concentrations induced a strong Ca^2+^-signal in all CGCs, indicating the presence of voltage-gated Ca^2+^ channels (VGCCs) (Fig. 4a, d). This finding fits well with the mature neuronal character of CGCs. Similar to high K^+^ concentrations, capsaicin elicited a robust Ca^2+^-signal in all CGCs, suggesting the uniform expression of TRPV1 which, to our knowledge, has not yet been described. In contrast, menthol and ATP never elicited a Ca^2+^-signal in CGCs, suggesting the absence of TRPM8 and P2X receptors.

To investigate whether the intracellular Ca^2+^-signal elicited by pH 6.0 in DAOY cells is ASIC-dependent, we used diminazene and PcTx1 (Fig. 4e). Both of these ASIC-inhibitors had no effect on the Ca^2+^-response, demonstrating that it was ASIC-independent. Similarly, in GSCs that also express ASIC1a, pH 6.0 does also not elicit Ca^2+^ signals [55]. Of note, the observed Ca^2+^-elevation required several seconds to be elicited and lasted up to several minutes, although a gradual amplitude decrease during stimulation was observed. Most likely, the ovarian cancer G protein-coupled receptor 1 (OGR1, GPR68), which has been previously characterized in DAOY cells [28], is responsible for the acid-induced Ca^2+^-signal in DAOY cells. OGR1 is an acid-sensitive G protein-coupled receptor (GPCR), which is expressed in a number of cancer cells and other cells associated with the tumor microenvironment (T-cells, macrophages, fibroblasts, endothelial cells) [60]. Therefore, it may be another therapeutic target in SHH MB.

### Extracellular acidification reduces cell viability but does not induce cell death in cultured MB cells

**Fig. 5:**
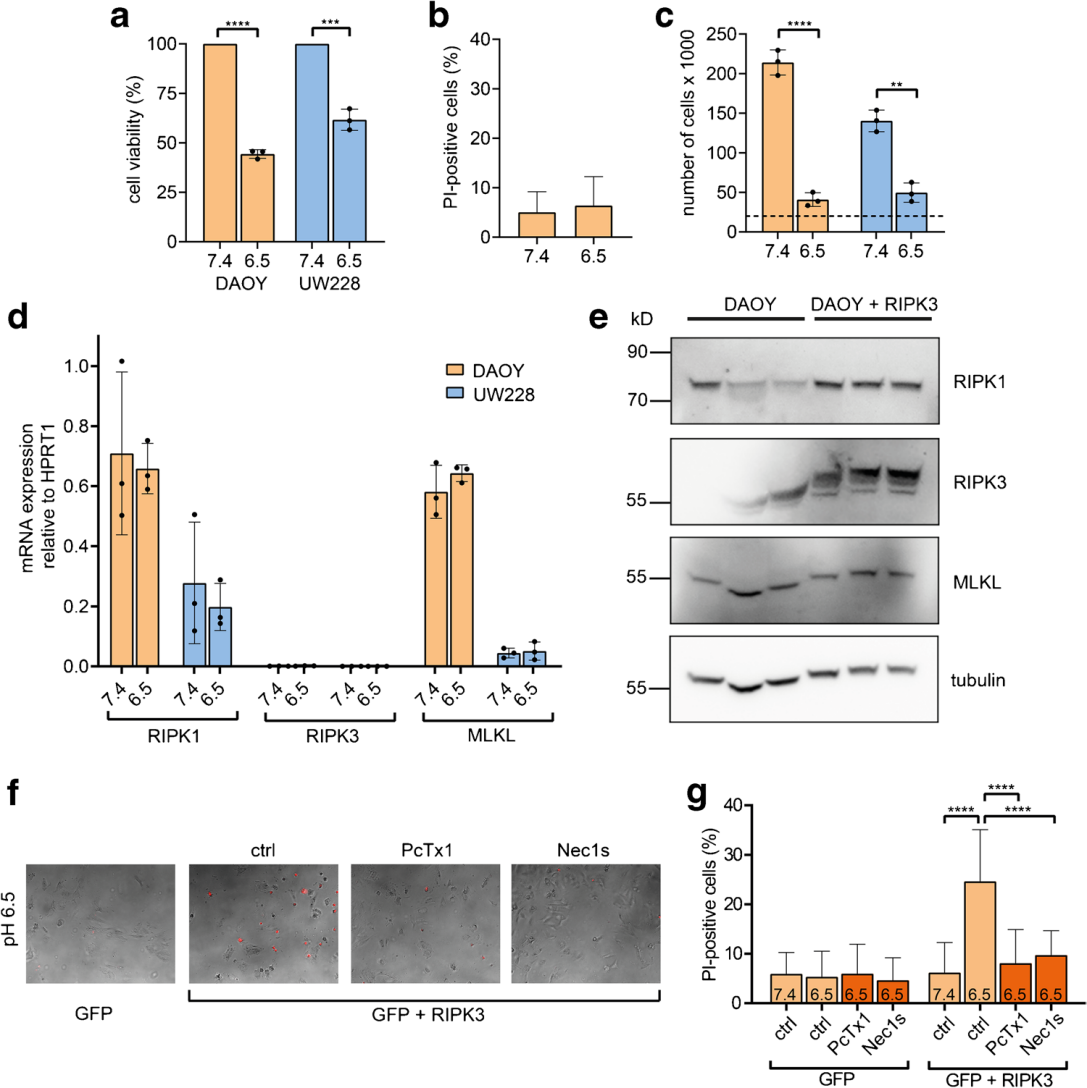
Overexpressing RIPK3 renders DAOY cells susceptible to acid-induced cell death. **a** Cell viability of DAOY and UW228 cells incubated for 24 h at pH 6.5 normalized to viability at pH 7.4 (*n* = 3). **b** Percentage of PI-positive DAOY cells at pH 7.4 (*n* = 28) and at pH 6.5 (*n* = 26). **c** Number of DAOY and UW228 cells after incubation for 72 h at pH 6.5 or at pH 7.4 (*n* = 3). The black dashed line indicates the number of cells (20,000) seeded at 0 h. **d** RNA expression of *RIPK1*, *RIPK3*, and *MLKL* in DAOY and UW228 cells incubated for 48 h at pH 7.4 or pH 6.5 (*n* = 3). **e** Immunoblot of untransfected DAOY cells (DAOY) or DAOY cells transfected with Flag-RIPK3 (DAOY + RIPK3), probed with antibodies against RIPK1, RIPK3, MLKL, and tubulin. Note the appearance of a higher molecular weight band for RIPK3 in DAOY cells transfected with RIPK3, suggesting that the RIPK3 signal in untransfected DAOY cells is unspecific. **f** Light microscopy images of DAOY cells after 48 h at pH 6.5 or at pH 7.4. The cells expressed either GFP or GFP plus RIPK3 and were treated with or without 100 nM PcTx1 or 20 μM Nec1s. PI-positive cells are in red. **g** Percentage of PI-positive DAOY cells (*n* = 30). Results are shown as mean + SD. Statistical analysis was performed with unpaired Student’s t-tests (**a**-**d**) or one-way ANOVA (**g**). **, *P* < 0.01; ***, *P* < 0.001; ****, *P* < 0.0001

Next, we determined whether ASICs influence cell viability and cell death under acidic conditions. We maintained DAOY and UW228 cells in neutral (pH 7.4) or slightly acidic (pH 6.5) medium for 24 h and measured cell viability with a CellTiter-Glo® assay; for DAOY cells, we also measured cell death with propidium iodide (PI) staining. In both DAOY and UW228 cells, acidic conditions strongly reduced viability (Fig. 5a), but did not cause an increase in cell death in DAOY cells (Fig. 5b). Since the reduction in viability was also present in UW228 cells, which do not express ASICs (Fig. 1), we hypothesize that this effect is ASIC-independent. Proliferation was also reduced at acidic pH (Fig. 5c). These findings are similar to the effect of acidic pH on GSCs [8]. In contrast, maintenance of mouse neurons in acidic medium for 24 h induces ASIC-mediated cell death [17, 52, 58, 59, 62]. It has been suggested that ASIC1a mediates cell death via a RIPK1-mediated, necroptosis-like pathway [58, 59]. We determined the mRNA expression of essential components of the necroptosis pathway, *RIPK1*, *RIPK3* and *MLKL*. DAOY cells robustly expressed *RIPK1* and *MLKL*, but almost no *RIPK3* (Fig. 5d). Similarly, UW228 cells also expressed almost no *RIPK3*, and only small amounts of *MLKL*. Expression of *RIPK1*, *RIPK3* and *MLKL* was not changed by maintaining DAOY or UW228 cells for 48 h at pH 6.5 (Fig. 5d). While we also detected RIPK1 and MLKL proteins by Western blot in DAOY cells, the presence of RIPK3 was ambiguous (Fig. 5e).

Therefore, we tested the hypothesis that acidic pH did not induce cell death in DAOY cells due to a downregulation of *RIPK3* and overexpressed RIPK3. RIPK3 could clearly be detected by Western blot in cells overexpressing RIPK3, but overexpression of RIPK3 did not induce cell death per se (Fig. 5g). In contrast, slight acidosis strongly increased cell death in DAOY cells expressing RIPK3 but not in GFP expressing DAOY cells (Fig. 5f, g). Strikingly, the increased cell death at acidic pH was abolished by the ASIC1a-inhibitor PcTx1 and by the RIPK1-inhibitor Nec1s. These results are consistent with the idea that DAOY cells are resistant to acid-induced cell death via down-regulation of *RIPK3* and that upon overexpression of RIPK3, DAOY cells become susceptible to ASIC1a-mediated cell death.

### Tissue analysis of pediatric patients from the GlioVis data portal reveals changes in the mRNA expression of ASIC subunits, RIPK1, RIPK3, and MLKL in intracranial tumors

**Fig. 6:**
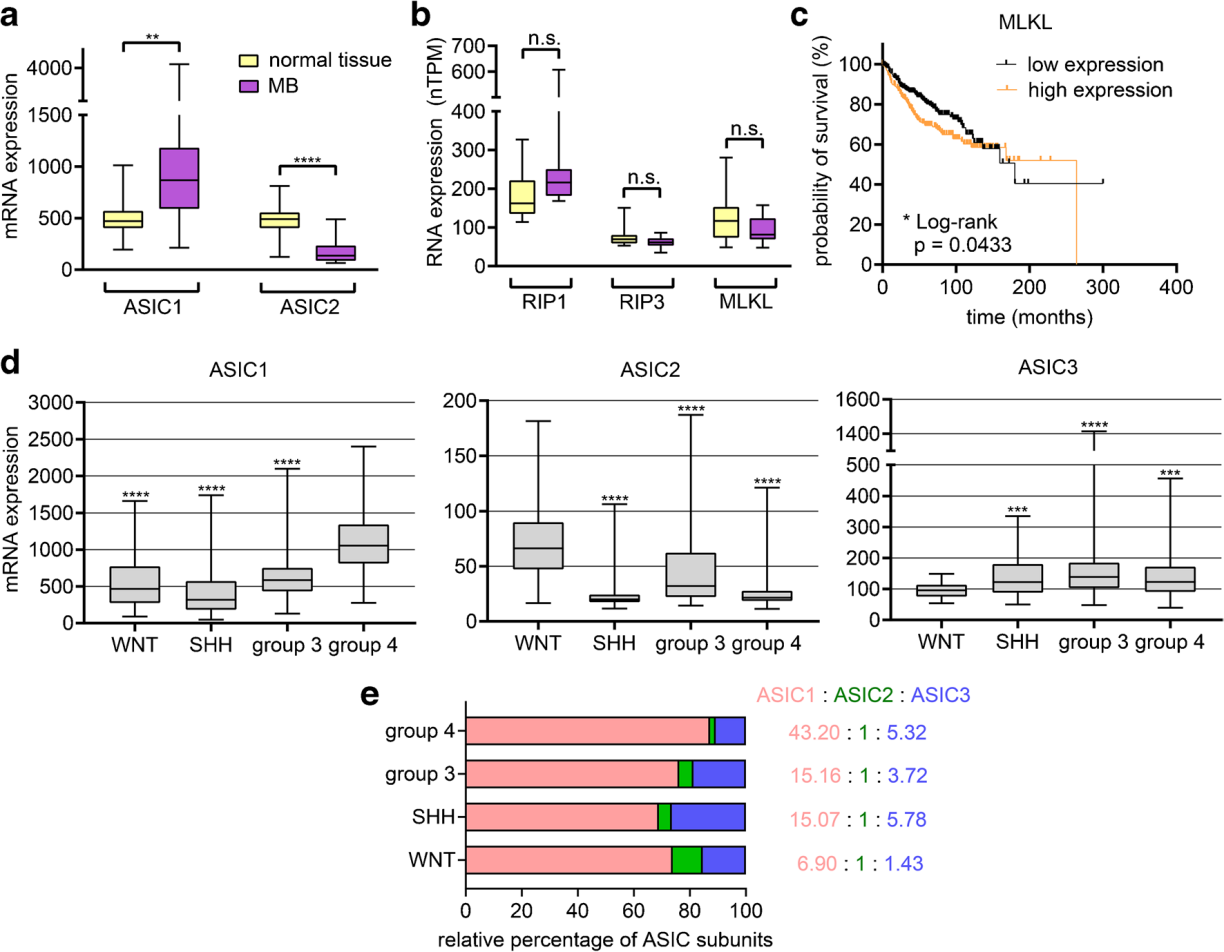
Expression of *ASIC1-ASIC3*, *RIPK1*, *RIPK3* and *MLKL* in MB and Kaplan–Meier survival analysis. **a**, **b** The Gump microarray dataset was used to determine the relative mRNA expression of *ASIC1* and *ASIC2* (**a**), and of *RIPK1*, *RIPK3*, and *MLKL* (**b**) in normal tissue (*n* = 16) and MB (*n* = 19) (n.s., no statistical significance). ASIC3 was not included in the dataset. Results are presented as box plots with whiskers showing the minimal and maximal values, respectively. **c** Kaplan–Meier survival curves of MB samples from the Cavalli microarray dataset with low (*n* = 312) or high (*n* = 320) mRNA expression of *MLKL* (above or below the median). **d** The Cavalli microarray dataset was used to determine the relative mRNA expression of *ASIC1-3* in the 4 molecular subgroups of MB (WNT: *n* = 70; SHH: *n* = 223; group 3: *n* = 144; group 4: *n* = 326). Results are presented as box plots with whiskers showing the minimal and maximal values, respectively. **e** Approximate *ASIC1*: *ASIC2*: *ASIC3* ratio in the 4 molecular subgroups of MB, based on the data in D. The microarray datasets were accessed on the 26.06.2022 through the GlioVis data portal for visualization and analysis of brain tumor expression datasets. Statistical analysis was performed with one-way ANOVAs (**a**, **b**, **d**) or a Log-rank (Mantel-Cox) test (**c**). **, *P* < 0.01; ***, *P* < 0.001; ****, *P* < 0.0001

To confirm expression of ASICs and components of the necroptosis pathway in human MB samples, we used two freely available microarray datasets from the GlioVis data portal (gliovis.bioinfo.cnio.es) [5]. The Gump microarray dataset revealed a significantly elevated expression of *ASIC1* in MB compared to normal tissue and a downregulation of *ASIC2* (Fig. 6a). *ASIC3* was not included in this dataset. Comparing the different molecular subgroups of MB using the Cavalli dataset (Fig. 6d), revealed that SHH tumors expressed the lowest relative amount of *ASIC1*, while group 4 tumors had the highest amount. *ASIC2* expression was lowest in SHH and group 4 tumors and highest in WNT tumors. *ASIC3* expression was lowest in WNT and highest in group 3 tumors. In all subgroups, expression of *ASIC1* was 5–15 times higher than that of *ASIC2* and *ASIC3*. Calculating the ASIC subunit ratios for the different MB subgroups (Fig. 6e) revealed that WNT MB, the most benign MB subtype, had an ASIC1:ASIC2 ratio of 7:1, which is similar to normal brain tissue [61], and that group 4 MB had a much higher ASIC1:ASIC2 ratio of 43:1. The ratios in SHH MB and group 3 MB were in between WNT and group 4 MB.

In contrast, *RIPK1*, *RIPK3*, and *MLKL* expression did not change in MB compared to normal tissue (Fig. 6b); *RIPK3* expression was substantially lower than *RIPK1* expression. Kaplan–Meier survival analyses of patients with low and high expression levels revealed no significant difference for *ASIC1*, *ASIC2*, *ASIC3*, *RIPK1*, and *RIPK3* but a significantly longer survival for patients with a low expression of *MLKL* (Fig. 6c).

## Discussion

Our study has three key findings. First, DAOY cells, a commonly used model for SHH MB, express functional ASICs, primarily homomeric ASIC1a. Second, the most numerous neurons in the brain, cerebellar granule cells, which have the same progenitor cells as SHH MB, also express primarily homomeric ASIC1a. Third, DAOY cells are resistant to acid-induced cell death and express almost no RIPK3; however, overexpression of RIPK3 renders them susceptible to acid-induced cell death via activation of ASIC1a.

qPCR analysis showed an approximately fivefold higher expression of *ASIC1* than of *ASIC2* in DAOY cells (Fig. 1a). Sensitivity of the ASIC current to PcTx1 confirmed the presence of homomeric ASIC1a (Fig. 1d). Although we cannot exclude some expression of heteromeric ASIC1a/2a or heteromeric ASIC1a/2b, our results suggest that DAOY cells primarily express homomeric ASIC1a. Interrogation of the GlioVis database revealed that MB tissue, specifically SHH MB tissue, compared with normal brain tissue, is characterized by an increased expression of ASIC1 and a reduced expression of ASIC2 (Fig. 6). Thus, the expression pattern of ASICs in DAOY cells is representative of SHH MB, indicating that DAOY cells are an appropriate model for studying the role of ASICs in SHH MB. In contrast, UW228 did not express ASIC mRNA and pH 6.0 did not elicit ASIC currents in these cells. The absence of ASICs in UW228 cells is not representative of fresh MB tissue, indicating that these cells are not a good model for studying the role of ASICs in MB. Interestingly, GSCs and GBM tissue also primarily express homomeric ASIC1a, whereas ASIC2a is downregulated [55], similar to DAOY cells and MB tissue. Moreover, it appears that a worse prognosis of a MB subgroup correlates with a high ASIC1/ASIC2 ratio. Whether this is a coincidence or whether a high ASIC1/ASIC2 ratio might be a cause for the poor prognosis of a tumor is currently unknown. Similarly, further research is necessary to determine the underlying pathophysiological mechanisms of the increased ASIC1/ASIC2 ratio.

qPCR and western blot analyses combined with electrophysiological characterization of ASIC currents consistently showed that mouse CGCs primarily express homomeric ASIC1 (Fig. 2). This finding is consistent with a biochemical analysis of mouse brains, which shows that in the mouse cerebellum,  > 75% of the ASIC protein is ASIC1a and < 5% is ASIC2a [61]. Although there is a substantial amount of ASIC2b protein (~ 20% of all ASICs), it is mostly not on the cell surface; thus, it is not expected to contribute to functional ASICs [61]. However, because ASIC1a homomers and ASIC1a/2b heteromers share very similar electrophysiological properties [51], we cannot exclude the contribution of heteromeric ASIC1a/2b to ASICs in CGCs. Previously, it had already been shown that ASIC currents in mouse CGCs are completely inhibited by 10 nM PcTx1 [16], and that genetic deletion of ASIC2 does not reduce ASIC currents in mouse CGCs [33], both results being consistent with a predominant role of homomeric ASIC1a in CGCs.

ASICs contribute to excitatory postsynaptic currents in the nucleus accumbens [37], lateral amygdala [14], and calyx of Held [23]. CGCs receive excitatory inputs from mossy fibers and inhibitory inputs from Golgi cells. Therefore, we speculate that ASICs contribute to postsynaptic currents at mossy fiber-CGC synapses. The ASICs of CGCs and DAOY cells were similar. Although we cannot exclude species differences between mouse and human cells, these results suggest that granule cell progenitors, from which SHH MB is derived [19], also express similar ASICs, primarily homomeric ASIC1a.

Several studies have shown that cultured neurons are sensitive to acid-induced cell death via ASIC1a activation [17, 52, 58, 59, 62]. It was, therefore, surprising that ASIC1a-expressing DAOY cells were resistant to acid-induced cell death. More recently, it has been shown that activation of ASIC1a induces a cell death pathway that depends on RIPK1 and is therefore likely related to necroptosis [58, 59]. This pathway is also active in GSCs where it depends on RIPK3 [8]; RIPK3 mRNA is not expressed in DAOY cells (Fig. 5d). Strikingly, RIPK3 expression was sufficient to render DAOY cells susceptible to acid-induced cell death. As cell death was inhibited by Nec-1 s and PcTx1 (Fig. 5f), it was RIPK1- and ASIC1a-dependent and likely related or identical to the acid-induced cell death pathway of neurons and GSCs. Interestingly, absence of RIPK3 expression is associated with necroptosis resistance in melanoma, and re-expression of RIPK3 is sufficient to render a set of melanoma cell lines susceptible to necroptosis [18]. In contrast to DAOY cells, MB tissue expresses RIPK3 (Fig. 6b). Because cells within MB tissue are heterogeneous, it is possible that some cells, such as DAOY, do not express RIPK3 and are therefore resistant to necroptosis-related cell death pathways. Alternatively, RIPK3 expression was downregulated in DAOY cells after their initial cultivation.

Is ASIC1a a potential new target for treating MB? At first glimpse, the induction of cell death via ASIC1a activation seems promising. However, the role of pro-inflammatory cell death pathways in cancer therapy is ambiguous [22, 47]. We indeed found that high expression of MLKL, the executioner protein of the necroptosis pathway, was associated with shorter survival (Fig. 6c), suggesting that necroptosis plays an unfavorable role in MB. Interestingly, the same is true for GBM [8, 13]. While the role of MLKL in cancer is complex and its pro- and anti-cancer effects depend on many parameters, including the respective cancer type and cellular microenvironment [44], it is not essential for acid-induced cell death in GSCs [8] and therefore perhaps also not in MB. Irrespective of these uncertainties, it is conceivable that acid-induced cell death contributes to the evolution of MB in its acidic TME in situ.

While ASICs were not involved in eliciting intracellular Ca^2+^ signals in DAOY cells, Ca^2+^ imaging suggested the presence of capsaicin-sensitive TRPV1, menthol-sensitive TRPM8, and ATP-sensitive P2X receptors in subgroups of DAOY cells (Fig. 4). Because it has been suggested that these ion channels are involved in the pathophysiology of many types of tumors [12, 40, 65], it would be interesting to determine in the future how they affect MB cells. Likewise, capsaicin elicited Ca^2+^ signals in all CGCs (Fig. 4). Given the emerging role of TRPV1 in a wide variety of CNS functions and pathologies, such as behavior, addiction, learning, and synaptic plasticity [27, 45], further studies of TRPV1 in CGCs would be of interest.

In summary, we provide compelling evidence for the expression of functional ASIC1a in MB and show that DAOY cells are a suitable model to study the role of ASICs in SHH MB. In addition, we show that, in principle, activation of ASIC1a can induce cell death in DAOY cells. They evade this cell death by an absence of RIPK3, providing further evidence for an ASIC1a-RIPK1-RIPK3 pathway in acid-induced cell death.

## Materials and methods

### Materials

PcTx1 (Smartox Biotechnology, Sainte-Égrève, France) and APETx2 (Alomone Labs, Jerusalem, Israel) were purchased in research quality.

### Cell culture of adherent DAOY, UW228, CHO cells and HEK-293 ASIC1a KO cells

DAOY cells and UW228 cells were kindly provided by G. Ciarimboli (Münster, Germany); UW288 cells were originally provided by John R. Silber (University of Washington, Seattle, USA). DAOY cells were cultured in Eagle’s minimal essential medium (MEM Eagle; PAN-Biotech, Aidenbach, Germany) supplemented with 10% fetal bovine serum (FBS; Sigma-Aldrich, St. Louis, MI, USA). UW228 cells were cultured in Dulbecco’s modified Eagle medium (DMEM)/F12 supplemented with 10% FBS and 2 mM L-Glutamine (Thermo Fisher Scientific). HEK-293 ASIC1a KO cells were kindly provided by S. Pless (Copenhagen, Denmark); they were cultured in DMEM supplemented with 10% FBS. CHO cells were cultured in DMEM/F12 supplemented with 10% FBS. Cells were maintained at 37 °C with 5% CO_2_ and passaged every 3–4 days with 0.25% (DAOY cells, UW228 cells) or 0.05% (HEK-293 ASIC1a KO cells, CHO cells) Trypsin–EDTA solution (Thermo Fisher Scientific).

### Cell culture of medullospheres

DAOY cells were cultured as medullospheres in suspension according to the protocol by Gong et al. [21]. First, adherent DAOY cells were allowed to become confluent in a 10 cm petri dish (Sarstedt AG & Co. KG, Nümbrecht, Germany). Next, they were detached with 0.25% Trypsin–EDTA, washed and centrifuged two times with DPBS (PAN-Biotech) and resuspended in medullosphere culturing medium. This medium contained DMEM/F-12 and the following supplements: 1% N-2 supplement (Gibco, Thermo Fisher Scientific), 2% B-27 supplement (PAN-Biotech), 20 ng/ml recombinant human fibroblast growth factor (FGF; 154 a.a.; Thermo Fisher Scientific) and 20 ng/ml recombinant human epidermal growth factor (EGF; R&D Systems, Minneapolis, USA). The cells were resuspended and transferred into a T75 Nunc™ non-treated flask (Thermo Fisher Scientific). Medullosphere culturing medium was added to the flask to a total of 20 ml. Cells were cultured for 7 d and then harvested for use in RT-qPCR.

### Isolation and maintenance of primary mouse cerebellar granule cells

For the Trypsin/DNAse solution, 100 mg Trypsin (Sigma-Aldrich) was mixed with 10 mg DNAse I (Sigma-Aldrich) and 100 μl MgCl (80 mM). Next, 9 ml HBSS (PAN-Biotech) were added and the pH was adjusted to 7.8. HBSS was added to a volume of 10 ml. For the DNAse I solution, 10 mg DNAse I was mixed with 50 mg glucose and 20 ml Neurobasal A medium (Gibco, Thermo Fisher Scientific). The solutions were sterile-filtered and stored in 1- or 2-ml aliquots at -80 °C.

Isolation of primary mouse CGCs was performed as described by Girbes et al. [20]. WT mice pups were euthanized at P7 via decapitation. The head was placed on ice. The skin and skull were removed with forceps and scissors, to expose the intact brain. After careful removal of bone and skin fragments around the cerebellum, the latter was positioned between the opened forceps and gently detached from the rest of the brain. The isolated cerebellum was put in ice-cold HBSS and cleaned carefully under a stereomicroscope (Stemi DV4, Carl Zeiss AG) from any foreign structures such as connective tissues and other brain stem structures. Afterwards, it was cut into 3 smaller pieces. The isolated pieces of the cerebella from different mice were transferred from the ice-cold HBSS to a clean 15 cm Falcon tube (Sarstedt AG & Co. KG). The pieces were washed 3 times with 5 ml ice-cold HBSS per 3 cerebella. HBSS was removed and 1 ml per 3 cerebella of a Trypsin/DNAse solution was added. The cerebella were incubated at RT for 15 min and then washed again with 5 ml ice-cold HBSS per 3 cerebella. HBSS was removed and 1 ml per 3 cerebella of a DNAse I solution was added. The pieces of the cerebella were mechanically homogenized with glass Pasteur pipettes with a continuously decreasing diameter. To test whether the cerebella were appropriately homogenized, they were put on ice for 5 min. If no precipitate was visible after 5 min, they were homogenized successfully into single cells. If a precipitate was visible, the homogenization steps were repeated. Next, the homogenized samples were centrifugated for 15 min at 100 g and 4 °C. The supernatant was aspirated and the neurons were resuspended in 5 ml medium X-1 per 3 cerebella (prewarmed to 37 °C). Finally, the number of neurons was determined with a Neubauer chamber. For RT-qPCR and patch clamp experiments, CGCs were seeded at a density of 2,000,000 cells/ml in coated 96-well plates (RT-qPCR) or in coated glass cover slips (patch clamp). Neurons were put in the incubator for at least 24 h before use in an experiment.

CGCs were cultured in Medium-X1. This medium consisted of Neurobasal A medium and the following supplements: 4 nM L-Thyroxine (Sigma-Aldrich), 30 nM sodium selenite (Sigma-Aldrich), 1 mM sodium pyruvate (Sigma-Aldrich), 100 μg/ml Bovine transferrin holo (Merck Millipore, Billerica, USA), 0.1% BSA (SERVA Electrophoresis GmbH, Heidelberg, Germany), 2% B-27, 1% Penicillin/streptomycin (Sigma-Aldrich), 2 mM L-Glutamine, 10 μg/ml human recombinant insulin (Thermo Fisher Scientific) and 25 mM KCl. After thoroughly mixing, the medium was filtered through 0.22 μm syringe filters (Corning Incorporated, Corning, USA). Prior to seeding, 96-well plates and glass cover slips were coated with 0.1% Poly-L-lysine (Sigma-Aldrich) for at least 30 min at 37 °C.

### Transfection

DAOY cells were seeded in a 6-well plate and allowed to grow at 80% confluency during 24 h. Next, they were transfected either with GFP or with GFP + human Flag3-RIPK3 plasmids using FugeneHD® transfection reagent (Active Motif, Carlsbad, USA) at a 2:5 plasmid:reagent ratio. After 24 h, transfection rates of > 70% were achieved. Cells were then harvested and used for PI-stainings.

HEK-293 ASIC1a KO cells were seeded in a 10 cm petri dish and allowed to grow at 80% confluency during 24 h. Next, they were transfected with ASIC1a, ASIC2a or ASIC3 plasmids using polyethylenimine (PEI) at a 1:3 plasmid: PEI ratio. After 24 h, transfection rates of > 50% were achieved. Cells were then harvested and used for western blot.

### Whole-cell patch clamp

DAOY cells were seeded on glass coverslips and allowed to attach for at least 1 h in the incubator. CGCs were seeded on coated cover slips and incubated for at least 24 h before recording. Next, the coverslips were mounted in a perfused bath on the stage of an inverted microscope (IX71, Olympus) and kept at RT. The conditioning bath solution contained (in mM): NaCl 115, KH_2_PO_4_ 0.4, K_2_HPO_4_ 1.6, D-glucose 5, HEPES 5, glucose 5.5, MgCl_2_ 1, sodium gluconate 25, calcium gluconate 3. pH was adjusted to 7.4 with NAOH and HCl. The acidic bath solutions contained the same chemicals as the conditioning bath solution, except for HEPES being exchanged by MES. Acidic pH was adjusted with NaOH or HCl.

Patch-clamp experiments were performed in the whole-cell configuration. Patch pipettes had an input resistance of 5–7 MΩ for DAOY cells and 8–10 MΩ for cerebellar granule cells, when filled with an intracellular-like solution containing (in mM): potassium gluconate 95, KCl 30, NaH_2_PO_4_ 1.2, Na_2_HPO_4_ 4.8, D-glucose 5, MgCl_2_ 2.38, EGTA 1, calcium gluconate 0.726; pH was adjusted to 7.2 with KOH and HCl. Currents were recorded using a patch-clamp amplifier (Axopatch 200 B), the Axon-CNS (Digidata 1440 A) and Clampex software (Molecular Devices). Data was filtered at 1 kHz with a low-pass filter and was analysed with the PCLAMP software. The sampling rate was 20 kHz.

### Ratiometric Ca^2+^ -imaging

To determine intracellular Ca^2+^-concentrations, DAOY cells were seeded on cover slips, mounted in a cell chamber and perfused as described in the section “Whole-cell patch clamp”. Fluorescence was measured every 2 s on an inverted microscope (IX71, Olympus, Chromaphor) using a Fluar 20 × /0.75 objective (Olympus) and Till Vision real-time imaging software (Till Photonics). Cells were loaded for 30 min at 37 °C with 2 μM Fura-2-AM (Molecular Probes) in the bath solution. Fura-2 was excited at 340/380 nm and the emission was recorded between 470 and 550 nm using a sensicam CCD camera (PCO imaging). Acquisition and data analysis were done using the Till Vision software and Excel.

The bath solutions used for the capsaicin, menthol, ATP and ionomycin stimulations consisted of the conditioning bath solution described under “Whole-cell patch clamp” containing the following chemicals: 100 μM capsaicin (Sigma Aldrich), 200 μM menthol (Sigma Aldrich), 100 μM ATP (Sigma Aldrich), or 1 μM ionomycin (Santa Cruz Biotechnology, Dallas, USA), respectively. The 30 mM K^+^ solution contained the following compounds (in mM): NaCl 85, KCl 30, D-glucose 5, HEPES 5, glucose 5.5, MgCl_2_ 1, sodium gluconate 25, calcium gluconate 3. pH was adjusted to 7.4 with NAOH and HCl. The pH 6.0 solution consisted of the acidic bath solution described in “Whole-cell patch clamp”.

### Propidium iodide staining

2,000 DAOY cells were seeded in each well of a 96-well plate. The cells were then cultured in different conditions for 24 h. pH 7.4 (pH 6.5) medium was made by adding 1.2 g/l (0.1725 g/l) NaHCO_3_ to NaHCO_3_-free powdered MEM (Gibco, Thermo Fisher Scientific). Nec-1 s was purchased from Santa Cruz Biotechnologies. After incubation for 24 h, 1 μg of propidium iodide (Thermo Fisher Scientific) was added to each well and cells were incubated for another 10 min. Using an inverted microscope (IX71, Olympus, Chromaphor) with a sensicam CCD camera (PCO imaging), transmission images from the cells were acquired. The cells were excited with green light (555 nM) and fluorescent images were acquired. The total number of cells was determined by counting the cells in the transmission images using ImageJ. The amount of PI-positive cells was determined by counting the cells in the fluorescent images. The relative number of PI-positive cells in % was then calculated by dividing the number of cells counted in the fluorescent images with the ones counted in the transmission images.

### Cell viability assay

2,000 DAOY or UW228 cells/well were seeded in a 96-well plate. The cells were then cultured in different conditions for 24 h. The media for DAOY cells were prepared as described under “Propidium iodide staining”. For UW228 cells, pH 7.4 (pH 6.5) medium was made by adding 1.2 g/l (0.3377 g/l) NaHCO_3_ to NaHCO_3_-free powdered DMEM/F-12 (Gibco, Thermo Fisher Scientific). Afterwards, a CellTiter-Glo® Luminescent Cell Viability Assay (Promega, Fitchburg, USA) was performed as follows: 50 μl of the assay reagent was added to each well containing 100 μl medium each. The wells were shaken gently for 2 min on an orbital shaker, then incubated at RT for 10 min. Luminescence was measured with an Orion II microplate luminometer (Berthold Technologies, Bad Wildbad, Germany).

### Proliferation assay

20,000 DAOY or UW228 cells/well were seeded in a 6-well plate. The cells were then cultured in different conditions for 72 h. The media for the DAOY and UW228 cells were prepared as described under “Cell viability assay”. Afterwards, cells were detached from the wells with 0.25% Trypsin, pipetted into 1.5 ml Eppendorf tubes and resuspended in 200–500 μl medium. Cells were then counted with a CASY TT cell counter (Roche Applied Science, Penzberg, Germany).

### Reverse transcription quantitative PCR

Adherent DAOY and UW228 cells were cultured for 48 h prior to RNA isolation. DAOY cells in suspension were cultured for 7 days in an MBS culture prior to RNA isolation. Mouse CGCs were cultured for 48 h after isolation prior to RNA isolation. Total RNA was isolated using NucleoSpin RNA isolation kit (Macherey–Nagel, Düren, Germany). Concentration and quality of the RNA was measured using a NanoDrop 2000c spectrophotometer (Thermo Fisher Scientific). RNAs with a 260 nm/280 nm ratio > 2.0 and a 260 nm/230 nm ratio > 1.8 were used for reverse transcription. RNA was reverse transcribed to cDNA using the High-Capacity cDNA Reverse Transcription Kit (Thermo Fisher Scientific).

For reverse transcription quantitative real-time PCR (RT-qPCR), each reaction contained 1 μl cDNA (20 ng cDNA in 1 μl H_2_O), 1 μl AM-MGB labelled hydrolysis probe (TaqMan™; Thermo Fisher Scientific), 5 μl Luna Universal qPCR Master Mix (New England Biolabs, Ipswich, USA) and 3 μl H_2_O. For human DAOY and UW288 cells, the following TaqMan™ probes were used: HPRT1 (Hs02800695), RIPK1 (Hs01041869), RIPK3 (Hs00179132), MLKL (Hs04188505), Sox2 (Hs01053049), Nestin (Hs00707120), ASIC1a (Hs00952802), ASIC2a/b (Hs00153756), ASIC3 (Hs00245092), ASIC4 (Hs00539823). For mouse CGCs, the following TaqMan™ probes were used: HPRT (Mm03024075), ASIC1a/b (Mm01305996), ASIC2a/b (Mm00475691), ASIC2b (Mm00475687), ASIC3 (Mm00805460), ASIC4 (Mm01259052). A sample without cDNA served as negative control. Each reaction was pipetted into 4-Strip 0.1 ml Tubes (STARLAB, Hamburg, Germany) and transferred to the Rotor-Gene Q thermocycler (QIAGEN, Hilden, Germany) for measurement. Reactions were performed in technical triplicates for each biological replicate, with technical duplicate negative controls for each TaqMan™ probe. qPCR was performed in a Rotor-Gene Q PCR cycler (Qiagen), starting with a denaturation phase (180 s, 95°), followed by 40 cycles of denaturation (30 s, 95 °C), annealing (20 s, 60 °C) and extension (20 s, 72 °C). Experiments were repeated with RNA isolated from *n* = 3 independent cell batches and analysed using the ΔΔCt method. Efficiency of the house keeping genes HPRT1 (DAOY, UW228) and HPRT (CGC) was determined by a standard curve and was close to 100%.

### Western blot

HEK-293 ASIC1a KO control cells transfected with different ASIC subunits were used as positive controls. CHO cells which do not express native ASIC1, ASIC2a and ASIC3 channels were used as a negative control. CGCs were cultured for 48 h after isolation from the mice and prior to lysis. All cells were lysed using 100 μl/ well of 6-well plate of ice-cold lysis buffer containing 1% Triton X-100 (Sigma Aldrich) and protein inhibitor cocktail (cOmplete; Sigma-Aldrich) in PBS for 15 min on ice with gentle rocking. The cells were then scrapped with a cell scraper, and centrifuged at 8.000 × g for 10 min. Pellets were discarded and the supernatants were collected as samples, and boiled for 5 min in 1 × Laemmli buffer for separation in 10% SDS–PAGE gel under reducing conditions. The proteins were then transferred to a 0.45 μm pore size PVDF membrane (#10600023, Cytiva), which was blocked by immersion in blocking solution containing 5% fat-free dry milk powder in TBS with 0.05% Tween (Sigma Aldrich), pH 7.5 (TBS–T). The membrane was incubated with primary antibody in blocking solution overnight 4 °C with gentle rocking, washed in TBS–T, and probed with HRP-conjugated secondary antibodies (1:10.000 in blocking solution) for 1 h. The following primary antibodies were used: anti-ASIC1 (#833501, Biolegend), anti-ASIC2a (#ASC-012, Alomone labs), anti-ASIC3 (#ASC-018, Alomone labs), anti-RIP3 (#ab56164, Abcam), anti-RIP1 (#51–6559, BD Pharmingen), anti-MLKL (#14993, Cell Signaling Technology), and anti-tubulin (Sigma, #T5168). The following secondary antibodies were used: goat anti-Mouse IgG HRP (# 62–6520, Invitrogen), or goat anti-Rabbit IgG HRP (# 31460, Invitrogen). After washing, 300 μl of SuperSignal™ West Pico PLUS Chemiluminescent Substrate (Thermo Fisher Scientific) was applied to the membrane, which was imaged using a chemiluminescence camera (Vilber, Marne-la-Vallée, France). Experiments were repeated with *n* = 2 independently isolated CGC lysates and *n* = 3 independently isolated DAOY cells, respectively.

### Statistical analysis

Data are reported as mean ± SD. Statistical analyses were performed in Microsoft Excel 2007 and Prism 9 (Version 9.5.1), with significance threshold set to *p* ≤ 0.05. We assumed normal distribution and used parametric tests, paired or unpaired Student’s t-tests or one-way ANOVAs, for analysis of data from RT-qPCR, Ca^2+^-imaging, proliferation assays, cell viability assays, PI-stainings and patch clamp experiments. GlioVis microarray datasets were analysed with a one-way ANOVA and a Log-rank (Mantel-Cox) test.

## Data Availability

Datasets generated and analysed during the course of this study are included in this published article. Additional information is available from the corresponding author upon reasonable request.
